# Efficacy and safety of palbociclib plus endocrine therapy in North American women with hormone receptor‐positive/human epidermal growth factor receptor 2‐negative metastatic breast cancer

**DOI:** 10.1111/tbj.13516

**Published:** 2019-08-25

**Authors:** Karen A. Gelmon, Massimo Cristofanilli, Hope S. Rugo, Angela M. DeMichele, Anil A. Joy, Aurelio Castrellon, Bethany Sleckman, Ave Mori, Kathy Puyana Theall, Dongrui R. Lu, Xin Huang, Eustratios Bananis, Richard S. Finn, Dennis J. Slamon

**Affiliations:** ^1^ BC Cancer Vancouver BC Canada; ^2^ Feinberg School of Medicine Robert H. Lurie Cancer Center of Northwestern University Chicago IL USA; ^3^ Diller Family Comprehensive Cancer Center University of California San Francisco Helen San Francisco CA USA; ^4^ Abramson Cancer Center University of Pennsylvania Philadelphia PA USA; ^5^ Cross Cancer Institute University of Alberta Edmonton AB Canada; ^6^ Breast Cancer Center Memorial Healthcare System Hollywood FL USA; ^7^ Mercy Hospital St. Louis St. Louis MO USA; ^8^ Pfizer Oncology Milan Italy; ^9^ Pfizer Oncology Cambridge MA USA; ^10^ Pfizer Inc La Jolla CA USA; ^11^ Pfizer Oncology New York NY USA; ^12^ David Geffen School of Medicine University of California Los Angeles Santa Monica CA USA

**Keywords:** CDK4/6 inhibitor, metastatic breast cancer, North America, palbociclib

## Abstract

Palbociclib is a cyclin‐dependent kinase 4/6 inhibitor indicated for treatment of hormone receptor‐positive/human epidermal growth factor receptor 2‐negative advanced breast cancer in combination with endocrine therapy. We investigated the efficacy and safety of palbociclib in patients enrolled in North America during two‐phase 3 trials: PALOMA‐2 (n = 267, data cutoff: May 31, 2017) and PALOMA‐3 (n = 240, data cutoffs: April 13, 2018, for overall survival, October 23, 2015, for all other outcomes). In PALOMA‐2, treatment‐naïve postmenopausal patients with advanced breast cancer were randomized 2:1 to palbociclib (125 mg/d; 3 weeks on/1 week off [3/1]) plus letrozole (2.5 mg/d, continuous) or placebo plus letrozole. In PALOMA‐3, patients who progressed on prior endocrine therapy were randomized 2:1 to palbociclib (125 mg/d; 3/1) plus fulvestrant (500 mg, per standard of care) or placebo plus fulvestrant; pre/perimenopausal patients received ovarian suppression with goserelin. Palbociclib plus endocrine therapy prolonged median progression‐free survival vs placebo plus endocrine therapy in North American patients (PALOMA‐2: 25.4 vs 13.7 months, hazard ratio, 0.54 [95% CI, 0.40–0.74], *P* < .0001; PALOMA‐3: 9.9 vs 3.5 months, hazard ratio, 0.52 [95% CI, 0.38–0.72], *P* < .0001). Objective response and clinical benefit response rates were greater with palbociclib vs placebo in North American patients in both trials. While overall survival data are not yet mature for PALOMA‐2, median overall survival was increased in PALOMA‐3 (32.0 vs 24.7 months, hazard ratio, 0.75 [95% CI, 0.53–1.04]), though this did not reach statistical significance (*P* = .0869). Safety profiles in North American patients were similar to those of the overall populations; neutropenia was the most common treatment‐emergent adverse event. No new safety signals were observed. In summary, palbociclib plus endocrine therapy is an effective treatment option for North American women with hormone receptor–positive/human epidermal growth factor receptor 2–negative advanced breast cancer.

## 1 INTRODUCTION

Breast cancer is the most common cancer diagnosis in women from developed countries.^1^, ^2^ Incidence and mortality vary across countries due partly to differences in screening, lifestyle, and effects of race and ethnicity on treatment response.^1^, ^3^, ^4^ Understanding regional differences is critical to optimizing care. In the United States (US), approximately 266,000 new cases of breast cancer and 41,000 deaths occur annually.^5^ For women with advanced breast cancer (ABC), 5‐year survival rates are only 27%.^5^

Approximately 60% to 70% of patients with ABC have hormone receptor‐positive, human epidermal growth factor receptor 2‐negative (HR+/HER2−) tumors.^6^, ^7^ In this setting, endocrine therapy (ET) is the preferred option for patients who are not symptomatic or in visceral crisis.^2^, ^8^ Recent advances have enabled the development of combination strategies to delay progression and limit resistance to monotherapy, including targeting the cyclin‐dependent kinase (CDK) 4/6 pathway.^9^, ^10^, ^11^, ^12^

Palbociclib is a CDK4/6 inhibitor indicated in combination with an aromatase inhibitor as first‐line treatment of ABC and in combination with fulvestrant for progressive disease following ET.^13^ Full approval was based on the multinational phase 3 PALOMA‐2 and PALOMA‐3 trials.^14^, ^15^ Since the accelerated approval of palbociclib in 2015—based on the phase 1/2 PALOMA‐1 study^16^, ^17^CDK4/6 inhibitors in combination with ET have become a preferred treatment option for HR+/HER2− ABC.^2^, ^8^

Because palbociclib was first approved in North America, clinical use has been most extensive in this region. Therefore, it is of interest to examine the safety and efficacy of palbociclib in North American patients. We analyzed this subgroup using updated data from the PALOMA‐2 and PALOMA‐3 studies.

## 2 MATERIALS AND METHODS

### 2.1 Study design and patients

Detailed methods for both studies were previously published and are summarized in the Supplementary Information.^14^, ^15^, ^18^ In PALOMA‐2, all patients were postmenopausal. In PALOMA‐3, patients were enrolled regardless of menopausal status.

### 2.2 Treatment

Patients were randomized (2:1) to receive palbociclib (125 mg/d, orally; 3 weeks on, 1 week off) or placebo plus letrozole (2.5 mg/d, orally; continuous) in PALOMA‐2 and to receive palbociclib plus fulvestrant (500 mg intramuscularly on days 1 and 15 of cycle 1; once every 28‐day cycle thereafter) or placebo plus fulvestrant in PALOMA‐3, in 4‐week cycles.

### 2.3 Data analyses

This analysis compared the efficacy of palbociclib plus ET with that of placebo plus ET in a subset of the intent‐to‐treat (ITT) population enrolled in the United States and Canada, regardless of ethnicity. The primary end point in both trials was progression‐free survival (PFS), defined as time from randomization to radiologically confirmed disease progression—based on Response Evaluation Criteria in Solid Tumors (RECIST), version 1.1—or death. Secondary end points included overall survival (OS), objective response rate (ORR, proportion of patients with confirmed complete response [CR] or partial response [PR] per RECIST), and clinical benefit response rate (CBR, confirmed CR, PR, or stable disease for ≥24 weeks). Incidence of adverse events (AEs) was determined, and severity was graded according to National Cancer Institute Common Terminology Criteria for Adverse Events (version 4.0). Data cutoff was May 31, 2017, for PALOMA‐2. For PALOMA‐3, data cutoffs were April 13, 2018, (OS) and October 23, 2015 (all other outcomes).

The Kaplan‐Meier method was used to estimate median PFS and OS, with corresponding 95% confidence intervals (CIs). The Cox proportional hazard model was used to estimate hazard ratios (HR). ORR and CBR were summarized in the ITT population with measurable disease at baseline, and corresponding 95% CIs were calculated. No adjustments were made for multiple testing.

## 3 RESULTS

### 3.1 Patient population

In PALOMA‐2, median duration of follow‐up was 38 months in the palbociclib plus letrozole group and 37 months in the placebo plus letrozole group. Of 267 patients enrolled in North America (40% of the total population), 74% were enrolled in the United States and 26% in Canada. In PALOMA‐3, median follow‐up for all end points but OS was 16 months in the palbociclib plus fulvestrant group and 15 months in the placebo plus fulvestrant group. Of 240 North American patients (46% of the total population), 84% were enrolled in the United States and 16% in Canada. Approximately 17% and 15% of patients in the palbociclib and placebo arms, respectively, were pre/perimenopausal. Baseline demographics and disease characteristics were similar across treatment arms within each study (Table 1). Sites of recurrence were similar between treatment arms within each study; the most common metastatic site was bone (Table 1).

**Table 1:** Baseline demographics and disease characteristics in North American patients

Characteristics	PALOMA‐2		PALOMA‐3	
	PAL + LET (n = 168)	PBO + LET (n = 99)	PAL + FUL (n = 158)	PBO + FUL (n = 82)
Age, median (range), y	60.0 (30–86)	61.0 (28–88)	57.5 (31–88)	59.5 (29–77)
Race, n (%)				
White	141 (83.9)	86 (86.9)	129 (81.6)	71 (86.6)
Black	7 (4.2)	3 (3.0)	11 (7.0)	7 (8.5)
Asian	12 (7.1)	7 (7.1)	13 (8.2)	3 (3.7)
Hispanic/Latino ethnicity, n (%)	16 (9.5)	7 (7.1)	13 (8.2)	9 (11.0)
Weight, median (range), kg	71.0 (45.0–156.8)	69.5 (45.8–124.8)	70.7 (44.9–121.7)	73.7 (47.2–126.8)
Measurable disease present, n (%)	127 (75.6)	79 (79.8)	120 (75.9)	67 (81.7)
Recurrence type, n (%)				
Locoregional	1 (<1.0)	2 (2.0)	6 (3.8)	7 (8.5)
Local	2 (1.2)	0	9 (5.7)	3 (3.7)
Regional	3 (1.8)	0	7 (4.4)	2 (2.4)
Distant	102 (60.7)	64 (64.6)	104 (65.8)	50 (61.0)
Newly diagnosed	60 (35.7)	33 (33.3)	30 (19.0)	18 (22.0)
Unknown	0	0	2 (1.3)	1 (1.2)
Missing	0	0	0	1 (1.2)
Number of involved disease sites, n (%)				
1	53 (31.5)	26 (26.3)	50 (31.6)	24 (29.3)
2	43 (25.6)	30 (30.3)	46 (29.1)	22 (26.8)
3	38 (22.6)	24 (24.2)	34 (21.5)	17 (20.7)
4	22 (13.1)	13 (13.1)	17 (10.8)	15 (18.3)
>4	12 (7.1)	6 (6.1)	10 (6.3)	3 (3.7)
Not reported	0	0	1 (0.6)	1 (1.2)
Disease site, n (%)				
Visceral	77 (45.8)	51 (51.5)	96 (60.8)	54 (65.9)
Nonvisceral	91 (54.2)	48 (48.5)	62 (39.2)	28 (34.1)
Breast	55 (32.7)	32 (32.3)	20 (12.7)	10 (12.2)
Bone	126 (75.0)	75 (75.8)	119 (75.3)	65 (79.3)
Liver	29 (17.3)	20 (20.2)	59 (37.3)	42 (51.2)
Lung	54 (32.1)	35 (35.4)	47 (29.7)	25 (30.5)
Lymph node	78 (46.4)	47 (47.5)	61 (38.6)	31 (37.8)
Prior surgeries, n (%)	121 (72.0)	73 (73.7)	130 (82.3)	64 (78.0)
Prior radiation therapies, n (%)	86 (51.2)	56 (56.6)	110 (69.6)	62 (75.6)
Prior systemic therapies, n (%)	100 (59.5)	62 (62.6)	158 (100.0)	82 (100.0)
Previous chemotherapy regimen for primary diagnosis, n (%)	78 (46.4)	52 (52.5)	114 (72.2)	60 (73.2)
Previous hormonal regimen for primary diagnosis, n (%)				
Any	94 (56.0)	60 (60.6)		
1			61 (38.6)	44 (53.7)
>1			97 (61.4)	38 (46.3)
Sensitivity to prior hormonal therapy, n (%)	NA	NA	131 (82.9)	65 (79.3)

FUL, fulvestrant; LET, letrozole; NA, not applicable; PAL, palbociclib; PBO, placebo.

### 3.2 Efficacy

**Figure 1:**
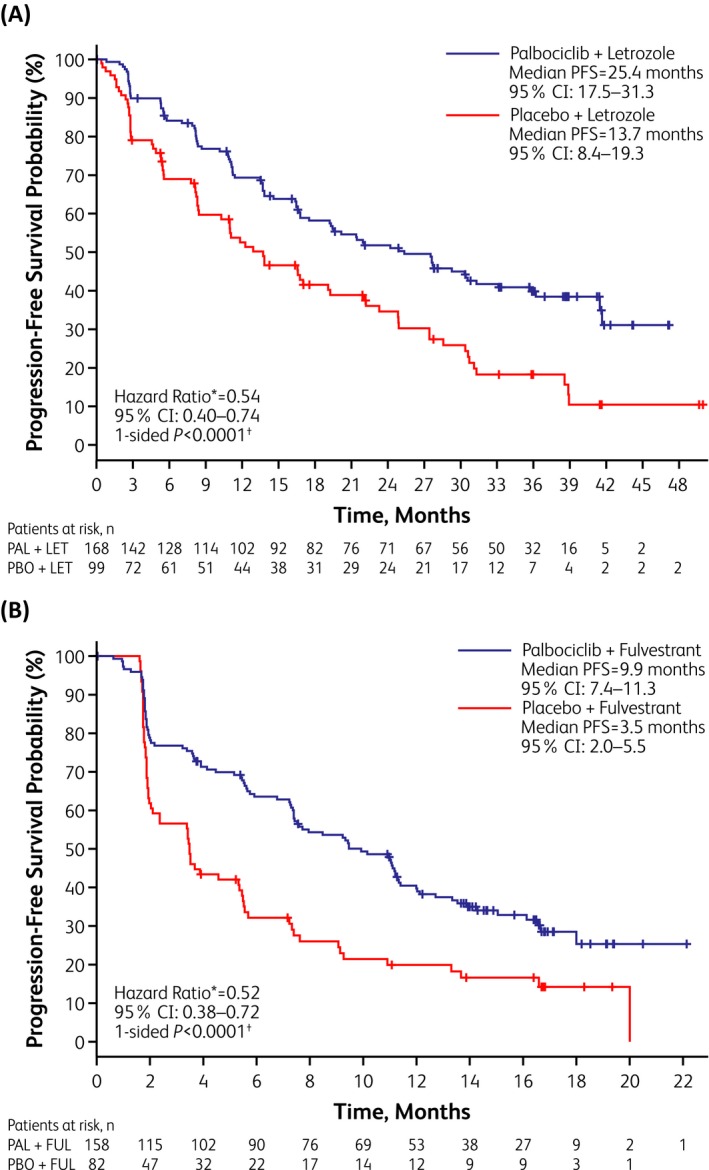
Investigator‐assessed progression‐free survival in North American patients. (A) Kaplan‐Meier curves for palbociclib plus letrozole vs placebo plus letrozole in PALOMA‐2. (B) Kaplan‐Meier curves for palbociclib plus fulvestrant vs placebo plus fulvestrant in PALOMA‐3. CI, confidence interval; FUL, fulvestrant; LET, letrozole; PAL, palbociclib; PBO, placebo. *Hazard ratio <1 indicates reduction in favor of PAL+LET/FUL. ^†^One‐sided, from log‐rank test

**Figure 2:**
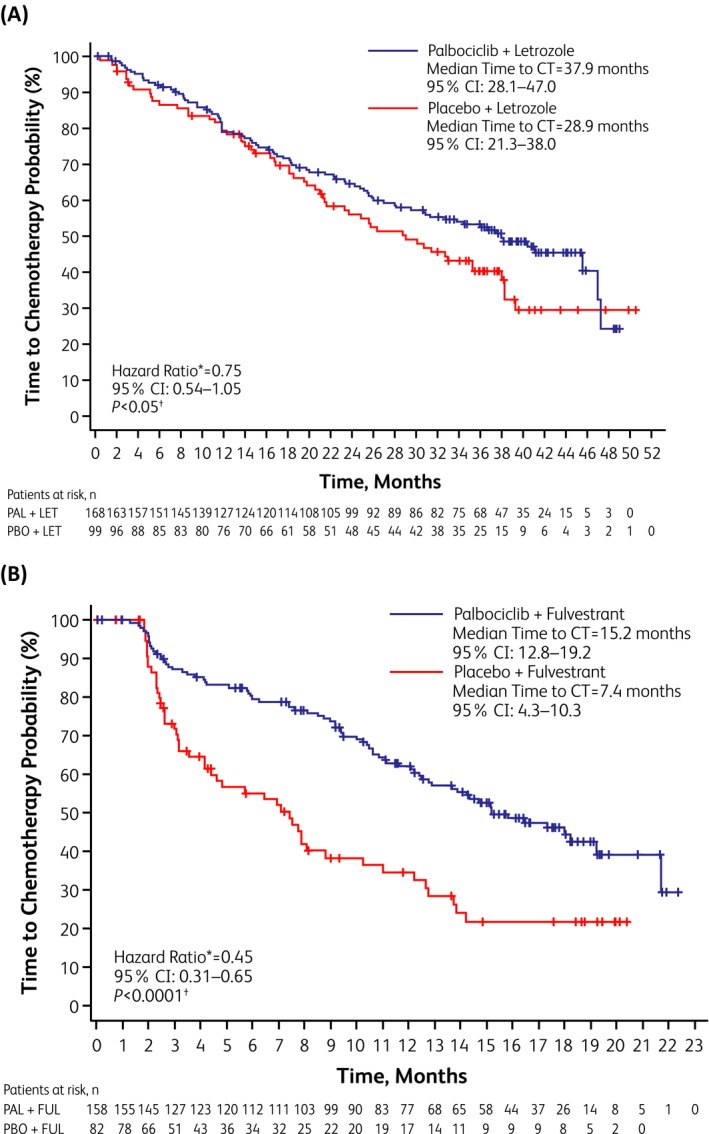
Time to first postprogression chemotherapy in North American patients. (A) Kaplan‐Meier curves for palbociclib plus letrozole vs placebo plus letrozole in PALOMA‐2. (B) Kaplan‐Meier curves for palbociclib plus fulvestrant vs placebo plus fulvestrant in PALOMA‐3. CI, confidence interval; CT, chemotherapy; FUL, fulvestrant; LET, letrozole; PAL, palbociclib; mo, month; PBO, placebo. *Assuming proportional hazards, hazard ratio <1 indicates reduction in favor of PAL+LET/FUL. ^†^One‐sided, from log‐rank test

In both studies, palbociclib plus ET prolonged PFS in North American women compared with placebo (Figure 1, Table 2). ORRs were higher in North American patients receiving palbociclib vs those receiving placebo in PALOMA‐2 (57% vs 52%) and PALOMA‐3 (24% vs 9%) (Table 2). North American patients in the palbociclib arms of both trials were also more likely to exhibit a CBR than patients in the placebo arms: 80% vs 67%, respectively, in PALOMA‐2 and 58% vs 28%, respectively, in PALOMA‐3. (Table 2). Data on OS in PALOMA‐2 were immature at the time of this analysis; however, updated data from the North American cohort of PALOMA‐3 (data cutoff, April 13, 2018) indicate that OS was longer with palbociclib than placebo (32.0 vs 24.7 months, HR, 0.75 [95% CI, 0.53–1.04]), although this difference did not achieve statistical significance (*P* = .0869) (Table 2). At the time of data cutoff, 76% of North American palbociclib‐treated patients in PALOMA‐2 and 71% of those in PALOMA‐3 had discontinued study treatment (vs 89% and 90% of placebo‐treated patients, respectively). Among North American patients, 38% of the palbociclib group and 51% of the placebo group in PALOMA‐2 received postprogression chemotherapy, with a median time to first chemotherapy of 37.9 and 28.9 months, respectively (Figure 2A). In PALOMA‐3, 46% of the palbociclib group and 61% of the placebo group received postprogression chemotherapy, with a median time to first chemotherapy of 15.2 and 7.4 months, respectively (Figure 2B).

**Table 2:** PFS, ORR, CBR, and OS in North American patients

	Median PFS, mo (95% CI)	PFS^a^, HR^b^ (95% CI); *P* Value^e^	ORR, %^d^ (95% CI)	ORR, OR^c^ (95% CI); *P* Value^e^	CBR, %^d^ (95% CI)	CBR, OR^c^ (95% CI); *P* Value^e^	Median OS, mo (95% CI)	OS, HR^b^ (95% CI); *P* Value^f^
PALOMA‐2								
PAL+LET	25.4 (17.5–31.3)	0.54 (0.40–0.74); *P* < .0001	57 (47.6‐65.5)	1.2 (0.7‐2.2); *P* = .2984	80 (72.3‐86.8)	2.0 (1.0‐4.0); *P* = .0250	NA	NA
PBO+LET	13.7 (8.4–19.3)		52 (40.4–63.3)		67 (55.6–77.3)		NA	
PALOMA‐3								
PAL+FUL	9.9 (7.4–11.3)	0.52 (0.38‐0.72); *P* < .0001	24 (16.8–32.8)	3.2 (1.2‐10.7); *P* = .0073	58 (48.1–66.5)	3.4 (1.7‐6.9); *P* = .0001	32.0 (27.6‐38.9)	0.75 (0.53‐1.04); *P* = .0869
PBO+FUL	3.5 (2.0–5.5)		9 (3.4–18.5)		28 (18.0–40.7)		24.7 (20.5‐31.0)	

Notes

a ITT population.

b Hazard ratio <1 indicates reduction in favor of PAL+LET/FUL.

c Odds ratio >1 indicates better response in favor of PAL+LET/FUL.

d ITT population with measurable disease.

One‐sided unstratified log‐rank test.

e One‐sided, from exact test.

f Two‐sided unstratified log‐rank test.

CBR, clinical benefit response rate; FUL, fulvestrant; HR, hazard ratio; ITT, intent‐to‐treat; LET, letrozole; NA, not available; OR, odds ratio; ORR, objective response rate; OS, overall survival; PAL, palbociclib; PBO, placebo; PFS, progression‐free survival.

### 3.3 Safety

Most North American patients experienced at least 1 AE (any grade) with palbociclib combination treatment (Table 3). The most common any‐grade and grade 3/4 AE in North American women in the palbociclib arm in both trials was neutropenia (Table 3). Febrile neutropenia was reported in 5 (3%) patients in the palbociclib arm (4 grade 3, 1 grade 4) in PALOMA‐2 and 1 (0.6%) patient (grade 3) in PALOMA‐3. Infections, fatigue, stomatitis, and alopecia, among others, were more common in palbociclib‐treated patients (Table 3). Increased alanine aminotransferase or aspartate aminotransferase occurred in 8.9% and 7.7% of patients, respectively, in PALOMA‐2 (vs 2% and 4% in the placebo group), and in 7.0% and 8.9% of patients, respectively, in PALOMA‐3 (vs 7.4% and 11.1% with placebo). Most increases were ≤2; <3% were grade 3. There were no elevations ≥grade 4, nor were there any reports of drug‐induced liver injury or hepatic failure.

**Table 3:** Common adverse events (≥20% in the palbociclib arm) in North American patients enrolled in PALOMA‐2 and ‐3 (as‐treated population)

Adverse event, %	PALOMA‐2				PALOMA‐3			
	PAL+LET (n = 168)		PBO+LET (n = 99)		PAL+FUL (n = 157)		PBO+FUL (n = 81)	
	All grades	Grades ≥3	All grades	Grades ≥3	All grades	Grades ≥3	All grades	Grades ≥3
Any adverse event	99.4	82.7	99.0	34.3	99.4	73.9	98.8	24.7
Neutropenia^a^	75.6	68.5	1.0	1.0	78.3	61.8	0	0
Infections^b^	67.9	8.9	52.5	5.0	51.0	1.3	33.3	2.5
Fatigue	59.8	4.8	44.4	1.0	57.3	2.5	42.0	2.5
Nausea	48.8	0	38.4	1.0	42.7	0	40.7	1.2
Arthralgia	46.4	1.8	43.4	2.0	23.6	1.3	23.5	0
Stomatitis^c^	36.9	2.4	20.2	0	30.6	1.3	14.8	0
Alopecia	36.3	0	19.2	0	21.7	0	9.9	0
Diarrhea	38.1	1.2	32.3	3.0	32.5	0	29.6	0
Hot flush	33.9	0	46.5	0	24.2	0	25.9	0
Headache	32.7	0.6	43.4	1.0	34.4	1.3	24.7	0
Leukopenia^d^	31.0	23.8	1.0	0	56.1	35.0	6.2	0
Back pain	31.5	1.8	31.3	0	19.7	1.9	17.3	2.5
Cough	32.1	0	25.3	0	23.6	0	19.8	0
Constipation	30.4	1.2	21.2	1.0	29.3	0	21.0	0
Vomiting	24.4	1.2	23.2	1.0	24.8	0	21.0	1.2
Dizziness	25.0	1.2	23.2	0	17.8	0.6	13.6	0
Insomnia	24.4	0	17.2	0	17.2	0.6	11.1	0
Pain in extremity	26.8	0.6	22.2	1.0	12.1	0.6	14.8	1.2
Upper respiratory tract infection	26.8	0	20.2	0	14.0	0	13.6	0
Rash^e^	25.0	1.2	17.2	1.0	19.1	0.6	8.6	0
Anemia^f^	22.6	7.7	5.1	1.0	29.9	3.2	14.8	1.2
Urinary tract infection	23.8	3.0	15.2	0	12.7	0	8.6	1.2
Thrombocytopenia	9.5	0.6	1.0	0	22.9	2.5	0	0

a Includes the Medical Dictionary for Regulatory Activities (MedDRA) preferred terms neutropenia and neutrophil count decreased.

b Includes the MedDRA preferred terms of system organ class infections and infestations.

c Includes the MedDRA preferred terms aphthous stomatitis, cheilitis, glossitis, glossodynia, mouth ulceration, mucosal inflammation, oral pain, oropharyngeal discomfort, oropharyngeal pain, and stomatitis.

d Includes the MedDRA preferred terms leukopenia and white blood cell count decreased.

e Includes the MedDRA preferred terms dermatitis, dermatitis acneiform, rash, rash erythematous, rash maculopapular, rash papular, rash pruritic, and toxic skin eruption.

f Includes the MedDRA preferred terms anemia, hematocrit decreased, and hemoglobin decreased.

FUL, fulvestrant; LET, letrozole; PAL, palbociclib; PBO, placebo.

In the North American cohort of PALOMA‐2, AE‐related dose reductions occurred in 73 (43.5%) patients in the palbociclib arm and 2 (2.0%) in the placebo arm. Dose interruptions or delays due to AEs occurred in 133 (79.2%) and 18 (18.2%) patients in the palbociclib and placebo arms, respectively. In PALOMA‐3, 56 (35.7%) patients in the palbociclib arm and 2 (2.5%) in the placebo arm had a dose reduction due to a treatment‐related AE. Dose interruptions or delays due to treatment‐related AEs occurred in 112 (71.3%) and 6 (7.4%) patients in the palbociclib and placebo arms, respectively. Permanent discontinuation of palbociclib or matching placebo treatment due to treatment‐emergent AEs occurred in 23 (13.7%) and 7 (7.1%) patients, respectively, in PALOMA‐2 and in 6 (3.8%) and 4 (4.9%) patients, respectively, in PALOMA‐3. Of note, only 2 (1.2%) North American patients in the palbociclib arm of PALOMA‐2 discontinued treatment due to treatment‐emergent neutropenia (1 grade 3, one grade 4); there were no neutropenia‐related treatment discontinuations in the placebo arm or in either treatment group in PALOMA‐3.

## 4 DISCUSSION

In the PALOMA‐2 and PALOMA‐3 trials, palbociclib plus ET prolonged PFS in the North American and overall populations (median PFS in PALOMA‐2: 25.4 and 27.6 months,^19^ respectively; PALOMA‐3: 9.9 and 11.2 months, respectively) and delayed treatment with cytotoxic chemotherapy. Updated data for North American patients from the PALOMA‐3 trial also indicated that OS was longer with palbociclib vs placebo (32.0 vs 24.7 months, HR, 0.75 [95% CI, 0.53–1.04]), *P* = .0869]), similar to results seen in the overall population (34.9 vs 28.0 months, HR, 0.81 [95% CI, 0.64–1.03]), *P* = .09]).^20^

Tumor response was similar with palbociclib in the overall and North American populations (Table 2),^21^, ^22^ providing further evidence that palbociclib plus ET leads to enhanced clinical benefit in North American patients with HR+/HER2− ABC. Of note, the magnitude of PFS benefit among European and Asian patients in the palbociclib arms of PALOMA‐2 (27.6 and 25.7 months, respectively) and PALOMA‐3 (13.4 and 12.9 months, respectively) was comparable to that in North American patients of diverse races and ethnicities, indicating that palbociclib has broad efficacy as both first‐ and later‐line treatment across geographic regions and ethnic groups.

The safety profile of palbociclib plus ET in North American patients was similar to that in the overall population. In both populations, the most common any‐grade and grade 3/4 AEs with palbociclib were hematologic (Table 3). Importantly, no new safety signals were observed in the North American population in either study at this later cutoff.

## 5 CONCLUSION

The present report is subject to several limitations, including its post hoc nature and small cohort size; moreover, analyses were not controlled for multiple comparisons. Nevertheless, these data suggest that palbociclib plus ET is a safe and effective treatment option for North American women with HR+/HER2− ABC who had not received prior systemic therapy for advanced disease or who progressed on prior ET. These findings are consistent with those seen in the overall ITT populations of both studies.
